# SCOWLP: a web-based database for detailed characterization and visualization of protein interfaces

**DOI:** 10.1186/1471-2105-7-104

**Published:** 2006-03-02

**Authors:** Joan Teyra, Andreas Doms, Michael Schroeder, M Teresa Pisabarro

**Affiliations:** 1 Department of Bioinformatics, BIOTEC TU Dresden, Tatzberg 47-51, 01307 Dresden, Germany

## Abstract

**Background:**

Currently there is a strong need for methods that help to obtain an accurate description of protein interfaces in order to be able to understand the principles that govern molecular recognition and protein function. Many of the recent efforts to computationally identify and characterize protein networks extract protein interaction information at atomic resolution from the PDB. However, they pay none or little attention to small protein ligands and solvent. They are key components and mediators of protein interactions and fundamental for a complete description of protein interfaces. Interactome profiling requires the development of computational tools to extract and analyze protein-protein, protein-ligand and detailed solvent interaction information from the PDB in an automatic and comparative fashion. Adding this information to the existing one on protein-protein interactions will allow us to better understand protein interaction networks and protein function.

**Description:**

SCOWLP (Structural Characterization Of Water, Ligands and Proteins) is a user-friendly and publicly accessible web-based relational database for detailed characterization and visualization of the PDB protein interfaces. The SCOWLP database includes proteins, peptidic-ligands and interface water molecules as descriptors of protein interfaces. It contains currently 74,907 protein interfaces and 2,093,976 residue-residue interactions formed by 60,664 structural units (protein domains and peptidic-ligands) and their interacting solvent.

The SCOWLP web-server allows detailed structural analysis and comparisons of protein interfaces at atomic level by text query of PDB codes and/or by navigating a SCOP-based tree. It includes a visualization tool to interactively display the interfaces and label interacting residues and interface solvent by atomic physicochemical properties. SCOWLP is automatically updated with every SCOP release.

**Conclusion:**

SCOWLP enriches substantially the description of protein interfaces by adding detailed interface information of peptidic-ligands and solvent to the existing protein-protein interaction databases. SCOWLP may be of interest to many structural bioinformaticians. It provides a platform for automatic global mapping of protein interfaces at atomic level, representing a useful tool for classification of protein interfaces, protein binding comparative studies, reconstruction of protein complexes and understanding protein networks. The web-server with the database and its additional summary tables used for our analysis are available at .

## Background

One of the most interesting and important challenges in the so-called "Post-genomic Era" is the understanding of protein networks. Protein-protein interactions have been extensively investigated using a variety of methods [1], and many databases have been built becoming very helpful tools for the analysis of protein networks [2-4].

Protein interfaces have long been studied at protein chain and domain interface levels [5-12]. Furthermore, numerous analyses have used datasets of protein chain interfaces to investigate residue type propensities, sequence and structure conservation at protein interfaces [8,11,13-16]. Databases containing structural domain-domain interactions have also been recently created: 3did [17], PiBase [18], iPfam [19], PSIbase [20], InterPare [21], PRISM [22]. However, in these methods still many protein residues are not taken into account as "interfacial" or "interacting" because of peptidic-ligands and also solvent being frequently ignored from the protein interaction analysis.

Peptidic-ligands and solvent mediate protein interactions and are fundamental components for a complete description of protein interfaces. Proteins can interact with peptides to perform their biological function. Besides, peptides have been used to mimic protein binding interfaces, and their complexes with proteins have been used to study protein binding affinity/specificity properties in a simplified way [23-25]. For these reasons, many protein-peptide complexes have been experimentally studied by X-ray crystallography and/or NMR studies, providing additional information on protein interfaces [25]. Moreover, protein interactions take place in an aqueous solution. Solvent molecules can bridge binding partners via hydrogen bonds contributing significantly to molecular recognition and function [23,26-31].

Most current methods do not provide an accurate description of protein interfaces, which is required to be able to establish the bases for understanding the principles that govern molecular recognition and protein function.

Here we present SCOWLP (Structural Characterization Of Water, Ligands and Proteins), a platform for complete and detailed characterization and visualization of protein interfaces. Our database includes all protein-interacting components of the PDB including peptides and solvent, which until now have been excluded from systematic protein interface analysis and databases. In our database all interface interactions are described at atom, residue and domain level by using interacting rules based on atomic physicochemical criteria. This complete characterization makes SCOWLP useful for comparative structural analysis of molecular interfaces. The web application allows the user to access all the atomic interaction information by querying the PDB or the SCOP hierarchy. All interface information characterized by different interaction descriptors can be interactively visualized by using a Jmol 3D applet [32].

## Construction and content

SCOWLP is a web-based relational database formed by eleven tables describing PDB interface interactions at atom, residue and domain level. The database contains 74,907 protein interfaces and 2,093,976 residue-residue interactions formed by 60,664 *structural units *and interacting solvent. For the creation of the SCOWLP, we extract 3D data of protein domains, peptidic-ligands and interface solvent from the PDB [33], and we define protein domains from the SCOP 1.69 [34]. We compute protein interactions at atom, residue and domain level by using bounding shape-based algorithms [35]. We also have developed a web application to handle and navigate through the interfacial data in an automatic and user-friendly fashion. We designed the SCOWLP methodology based on the following steps:

### SCOL-*Ligand *(*Structural Characterization Of Peptidic-Ligands*)

**Figure 1:**
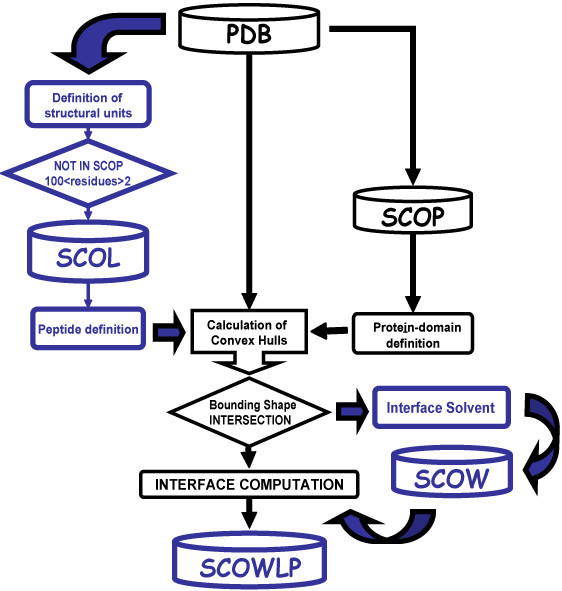
**Schematic overview of the methodology**. SCOWLP uses information from PDB, SCOP, SCOL and SCOW for the computation of atomic interface interactions.

**Figure 2:**
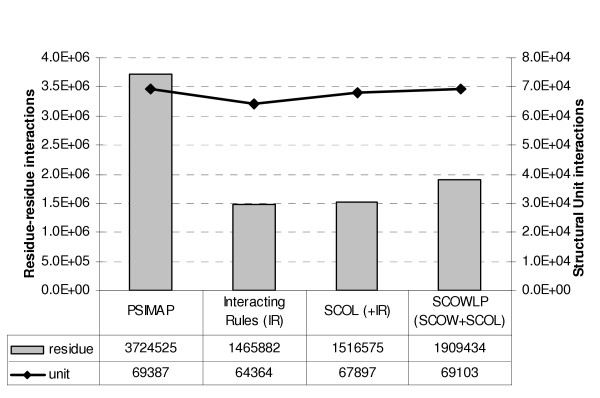
**Comparative histogram of SCOWLP vs. PSIMAP database**. Representation of the number of residue-residue (left *y *axis) and *structural unit *interactions (right *y *axis) contained in SCOWLP and comparison with PSIMAP.

The first step of our methodology consists of creating the SCOL table. Each *structural unit *in a PDB file is represented by a different chain name. We extract all *structural units *of the PDB and compare them with the domain definitions of SCOP. Although SCOP has a "Peptide" class containing functional peptides, it does not contain all peptidic-ligands complexed in the PDB. For this reason, *structural units *bigger than two and smaller than one hundred residues not defined in SCOP are considered *peptidic-ligands*. We stored this information in the SCOL table (Fig. 1). Heteroatoms and modified residues that form part of the same polypeptide chain are included, and DNA residues are excluded. We characterize each SCOL *peptidic-ligand *by resolution, sequence length and secondary structure. SCOWLP contains 2,739 peptidic-ligands, which add 3,413 new interfaces (Fig. 2).

### Interacting structural unit pairs

We label all *structural units *of the PDB with the SCOL-peptide and the SCOP-domain definitions in order to compute their interactions. We consider a contact distance cut-off of 9Å between two residues in order to allow up to two bridging water molecules in the shortest axes defining the interface. We use bounding shape-based algorithms to compute a 9Å convex hull (the smallest convex set containing all atoms at 9Å) for each *structural unit *of each PDB entry. Convex hull algorithms have been proved to reduce the computational time required for an interface calculation by both, reducing the search space to decrease the number of residues checked for the calculation and allowing distributed computations [35]. *Structural units *with intersecting shapes and having at least one residue-residue interaction are considered *interacting pairs *(Fig. 1).

### SCOW-*Water *(*Structural Characterization Of Water*)

**Figure 3:**
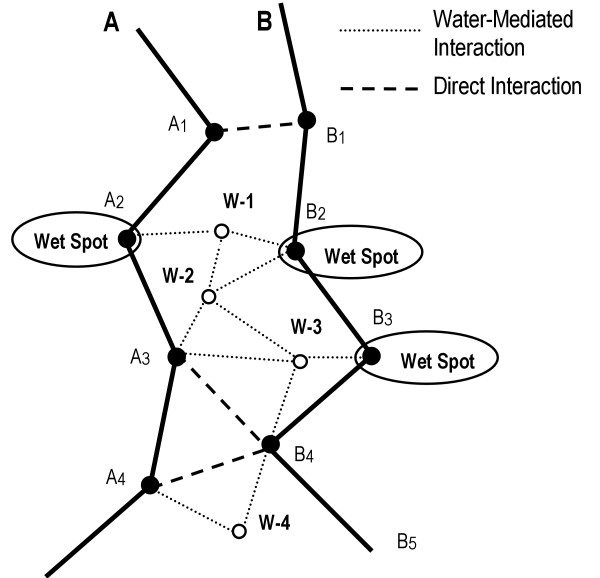
**Schematic representation of the interface interaction of two molecules and definition of *wet spots***. Molecules A and B form an interface. Interacting residues and water molecules are represented as black and open circles, respectively.

We consider a water molecule as part of an interface when it is located in the shape intersection of two interacting *structural units*. All interface water molecules are stored in the SCOW table and are then included in the atomic interface computation. We also consider an interaction when two residues are bridging through one or two water molecules. Residue contacts are defined as only water-mediated (*OWM*), non water-mediated or direct (*D*), and mixed (*M*). Residues that only interact through water are defined as *wet spots *(Fig. 3). SCOWLP contains 435,086 new water-mediated interactions thanks to the implementation of SCOW. This represents 20% of the SCOWLP database (Fig. 2).

### Interaction rules for interface computation

Only amino acid residues and water molecules placed in the intersection of *structural unit *shapes are potential interactors. We apply atom type and distance criteria to compute interactions between *structural unit *pairs at physicochemical level. For hydrogen bonds we apply a ≤ 3.2 Å donor-acceptor distance. For salt bridges, we apply a ≤ 4 Å distance criteria. Van der Waals energies are defined by hydrophobic atoms at van der Waals radii distance. At atomic level, we characterize the interactions by: i) nature: hydrophilic, hydrophobic; ii) contact type: main chain, side chain, mixed; iii) number of bridging water molecules. At residue level, we characterize the interactions by: i) nature: hydrophilic, hydrophobic, dual; ii) contact type: main chain, side chain, mixed; iii) number of bridging water molecules; iv) total number of atoms contacting. At *structural unit *level, we characterize the interactions by: i) contact volume; ii) surface area from convex hull surface; ii) number of interacting atoms/residues per unit; iv) type of interaction: intra-/inter-molecular. All interfacial interaction information is stored in the SCOWLP database (Fig. 1).

### Summary Tables

We have created the following additional tables for the filtering and comparative analysis of the information contained in the database:

#### Interface description

This table summarizes all interfaces of the SCOWLP database. It contains 74,907 interfaces constituted by SCOP domains labelled with the attributes: PDB Id code, atomic resolution, contact type (intra-/inter-molecular) and SCOP Id code. All interfaces are also labelled by number of interactions (total, all water-mediated and only water-mediated) and number of interacting residues per binding partner. Each interaction is classified by type (side-/main-chain or both) and by number of bridging water molecules.

#### Wet interfaces selection

This table stores interfaces of complexes at resolution ≤ 2.5 Å from the *Interface description table *for interfacial solvent analysis. This table does not include homodimer interfaces because of their patchy, poorly packed and highly hydrated nature [36]. With the resultant dataset, we create three tables:

##### Content

This table can be used to rank superfamilies based on their content in water mediating interface interactions. For each interface, it contains the average of total interactions, all water-mediated interactions and the ratio from the percentage of water-mediated interactions at superfamily level.

##### Morphology

This table can be used to rank the interfaces by number of *wet spots*. In this table each family is represented by the complex with the highest number of *wet spots*, labelled with the total number of interacting residues and *wet spots*.

##### Comparative

This table can be used to monitor solvent variations in interfaces and compare them at family level. It contains interfaces sorted out by domain, and then by their respective ligands (protein or peptide). Because a protein-ligand interface can be found in different PDBs, we select the interfaces that appear more than once and contain *wet spots*. When the same PDB file contains a repeated interface of two binding partners, we select as a representative the one with more *wet spots*.

### Implementation

We used MySQL and the Java programming language to generate and analyze the SCOWLP database. Interface calculations are performed on a 2.6 GHz Pentium IV in approximately 36 hours. SCOWLP is automatically updated with every SCOP release.

## Utility and Discussion

SCOWLP database contains detailed information of protein interfaces including peptidic-ligands and solvent in the PDBs, and classifies protein interfaces by using specific physicochemical atomic criteria. The database can be accessed through a user-friendly web application.

### Interaction rules

The use of atom type and distance rules allows us to characterize and classify interactions at physicochemical level. Other existing methods adopt exclusively a general distance criterion. PSIMAP [35], for example, considers as an interacting pair any atom distance at ≤ 5 Å. For this reason, the total number of residue-residue and *structural unit *interactions we obtain by applying our interaction rules is reduced in comparison to PSIMAP (Fig. 2). This reduction translates into more accurate interface definitions.

### Peptidic-ligand contribution

Some proteins have been subject of many structural studies complexed with peptides (e.g. Proteases, b.47.1). Besides, the superfamilies that have the higher occurrence of peptides are not necessarily those with higher domain-domain representation (e.g. Cyclophilin, b.62.1). By taking into account information about protein-peptide complexes SCOWLP contributes interfacial information of 8 SCOP superfamilies uniquely represented by protein-peptide complexes (a23.4, a.50.1, d.76.1, a.8.5, d195.1, g.33.1, a. 144.1, a. 12.1). In addition, it contributes with more than 50% of the interacting information in other superfamilies. Our results show the importance of including protein-peptide interfacial information in order to enrich considerably the description of protein interfaces.

**Figure 4:**
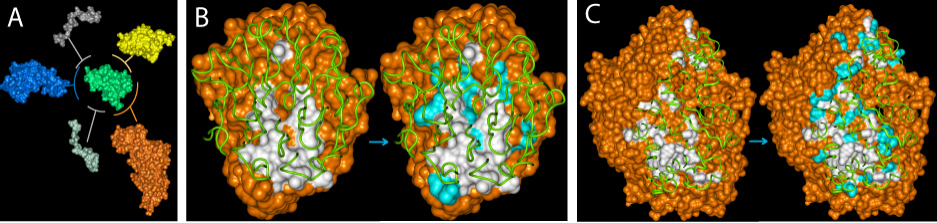
**Enrichment of the interface definitions by peptidic-ligands and solvent**. **A) **Enrichment in the description of protein interfaces by peptidic-ligands. The molecular recognition features of the BTB/POZ domain family are summarized. A representative POZ domain (green) is surrounded by five different ligands representing all possible BTB/POZ binding zones. Peptidic-ligands are represented in grey (PDB codes and chains used: 11dk_A, 11qb_ACD, 1r2b_D). **B) **Enrichment in the description of protein interfaces by *wet spots*. The complex of TEM1 β-lactamase (orange surface) with the inhibitor BLIPII (green ribbon; PDB 1jtd) is shown. White represents residues forming the interface before taking into account *wet spots *(in blue). **C) **The α and β (orange and green ribbon, respectively) chains of the Respiratory nitrate-reductase 1 (PDB 1q16) are shown. White represents residues forming the interface before taking into account *wet spots *(in blue). Figures created with *InsightII*, Accelrys.

Proteins can bind to peptides in places that do not exactly correspond to binding sites in their known protein-protein complexes. As an example, we show the BTB/POZ (Poxvirus and Zinc finger) family. The twelve BTB/POZ complexes in the PDB present five domain-binding regions, two of them described by the protein-peptide complexes (Fig. 4A). The POZ-peptide interfacial information is functionally relevant. It may help to propose new POZ contacts when reconstructing multi-protein complexes and modelling signalling pathways where the POZ domain-containing proteins are involved. Our results show that the addition of peptidic information can help to complete the view on how a protein recognizes its binding partners.

### Solvent contribution

All superfamiles of the *Content table *contain solvent mediating interactions. Furthermore, in some of these superfamilies water-mediated interactions represent up to 75% of the total interfacial interactions (e.g. d.250.1). Relating to the "only water-mediated" interactions, we observe from the *Morphology table *that 43 is the maximum number of *wet spots *found. Figures 4B and 4C illustrate how solvent, in particular *wet spots*, may play an important role in the morphological description of protein interfaces (shape and size). Considering the solvent, a discontinuous surface formed by several small isolated patches changes to a bigger and rounded patch. These observations show that we can enrich the description of protein interfaces by considering interfacial solvent.

Although solvent molecules mediating protein interactions can be conserved in a protein family, variations may occur due to different facts: i) atomic resolution and/or quality of the structural data, ii) conformational changes upon ligand binding, iii) protein flexibility, iv) new interacting regions (e.g. loop insertions and deletions), v) residue mimicry. *Wet spots *variations may be used as indicators in these cases. The *Comparative table *allows us to compare the interfaces of 127 families in 751 complexes based on *wet spots *variations.

Solvent molecules play an important role in the replacement of residues in protein interfaces. Sometimes the atomic resolution, the existence of different rotamers or even small differences in contact distances defining the interaction may influence the number of *wet spots*. Nevertheless, small variations of *wet spots *in complexes of the same family that do not present changes in *total number of interactions *can be used to locate residue mimicry cases (e.g. Lys+H2O≈Arg). Making use of this information may be very useful in analysis of protein interfacial evolution and in protein engineering/rational design when designing affinity and specificity of a protein for its ligands.

### Web application

SCOWLP contains atomic interfacial information of all the PDB entries structured by the SCOP hierarchy. There are two ways to query our database: SCOP or PDB. The user can query SCOP by keywords, SCOP/PDB Ids, or by simply navigating the SCOP hierarchical tree (Fig 5.1). When the user selects a family from the tree (labelled as FA), SCOWLP retrieves a list of the PDBs containing interfaces of that family in one frame. A second frame shows a summary table listing all the interfaces of that family with PDB id, type of contact, superfamily description of binding partners, interfacial area, total interacting residues and number of *wet spots*. This summary table gives a good overview over the interacting partners and interfacial variations at family level. By selecting any of the PDB IDs in this table, the user retrieves a list of all the interfaces of that PDB organized in two interactive tables: *Interfaces *and *Interactions*. We obtain the same tables querying SCOWLP by PDB ID (Fig 5.2). The "Interfaces" table shows binding partners, interfacial area, total number of interfacial residues and *wet spots*. The *Interaction Types *table classifies the interactions based on their water mediation, nature and type. The user can select the interfaces in a master/slave way to display a 3D molecular viewer and the selected domain contacts. We have implemented Jmol scripts [32] to allow the user to display and interactively analyze interfaces by using two control panels (Fig 5.3). The first one (on the right; Fig 5.3a; *Domain Contact Selection*) controls the interface display in the 3D viewer, allowing the user to highlight the residues forming part of each interface. The second panel (bottom left; Fig 5.3.c) controls: *Molecule View*: ON/OFF residue labelling, water mediators and spinning; *Interacting Descriptions*: interfacial residues colouring based on *wet spots*, nature and type. Fig. 5.3 shows a protein domain (red) interacting with a peptidic-ligand (yellow) and their respective interacting residues (*wet spots *in blue).

By using SCOWLP, the user can achieve specific queries, SCOP family analysis, interface comparisons and a detailed 3D display of the atomic interaction data contained in PDBs.

## Conclusion

Detailed analysis of the interfacial information contained in the PDB is very useful to obtain more accurate descriptions of protein interfaces. We have created SCOWLP to have a platform for the characterization and 3D visualization of protein interfaces. SCOWLP enlarges the available information on protein-protein interactions by introducing 3,413 new protein-peptide interfaces and 435,086 additional water-mediated interactions. All interactions contained in SCOWLP are characterized and classified at physicochemical level instead of using general distance criteria. This allows a more appropriate definition and enhanced comparison of the interfaces contained in our database.

As the origin of specificity and affinity in molecular recognition can be partially explained in terms of solvent's contribution to the interaction, our database constitutes a very useful tool to facilitate rational ligand design. In particular *wet spots *can be used as indicators of interfacial solvent variations, being helpful in comparison of protein family interfaces, and perhaps guiding docking experiments.

SCOWLP may be of interest to many structural bioinformaticians, representing a useful tool for classification of protein interfaces, protein binding comparative studies, reconstruction of protein complexes and understanding protein networks.

## Availability and requirements

SCOWLP is available at . The database and all summary tables used in this paper can be freely downloaded for independent studies.

## Authors' contributions

JT developed the new methodology to create the SCOWLP database. JT and AD designed the web application. MS provided the PSIMAP software code. MTP coordinated and supervised the project.

## Figures and Tables

**Figure 5:**
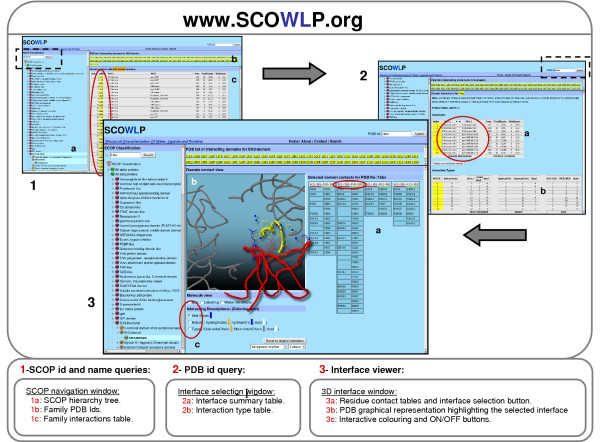
Screenshots and legends showing the structure of the SCOWLP website.
